# Acute interstitial nephritis with acetaminophen and alcohol intoxication

**DOI:** 10.1186/1824-7288-37-17

**Published:** 2011-04-15

**Authors:** Lauren L Fruchter, Iakovina Alexopoulou, Keith K Lau

**Affiliations:** 1 Faculty of Health Sciences, McMaster University, Ontario, Canada; 2 Department of Pathology, McMaster University, Ontario, Canada; 3 Department of Pediatrics, McMaster University, Ontario, Canada

**Keywords:** drug induced hypersensitivity, acetaminophen, renal injury, acute interstitial nephritis, alcohol

## Abstract

Drug-induced acute interstitial nephritis (AIN) represents a growing cause of renal failure in current medical practice. While antimicrobials and non-steroidal anti-inflammatory drugs are typically associated with drug-induced AIN, few reports have been made on the involvement of other analgesics. We report our experience in managing a 17-year-old female with AIN and subsequent renal injury following an acetaminophen overdose in conjunction with acute alcohol intoxication. It is well established that acetaminophen metabolism, particularly at high doses, produces reactive metabolites that may induce renal and hepatic toxicity. It is also plausible however, that such reactive species could instead alter renal peptide immunogenicity, thereby inducing AIN. In the following report, we review a possible mechanism for the acetaminophen-induced AIN observed in our patient and also discuss the potential involvement of acute alcohol ingestion in disease onset. The objective of our report is to increase awareness of healthcare professionals to the potential involvement of these commonly used agents in AIN pathogenesis.

## Introduction

Drug-induced acute interstitial nephritis (AIN) represents a common cause of renal injury in today's medical practice. AIN is characterized by cellular infiltration of the interstitium (including CD4+ T cells, macrophages, eosinophils and plasma cells) and is accompanied by interstitial edema in the absence of glomerular or capillary damage. The majority of AIN cases have been linked to the use of antimicrobials and non-steroidal anti-inflammatory drugs (NSAIDs); however the number of drugs implemented in AIN pathogenesis continues to expand [1,2].

Animal models of drug-induced AIN provide evidence for an immune-mediated mechanism of disease. On the basis of observed cellular infiltrate composition and physiological signs (fever, malaise, rash, eosinophilia and arthralgia), a T-cell mediated delayed hypersensitivity reaction has been proposed [1,3]. In this model, drugs are assumed to elicit reactions targeted to endogenous renal antigens through a hapten-like manner or through native renal protein imitation [1,2].

The following report describes a patient who experienced AIN after consumption of high doses of acetaminophen with concomitant acute alcohol intoxication. While hepatic and renal toxicity have been well associated with acetaminophen overdose [4], few reports have been documented on the involvement of the analgesic in AIN onset. Of the few cases reported, most patients, if not all, have also suffered from chronic alcoholism [5]. The documentation of AIN associated with acetaminophen overdose and acute alcohol ingestion is unprecedented. Thus, the purpose of our report is to increase awareness of the potential adverse affects of acetaminophen overdose; even in the case of acute alcohol consumption.

## Case Report

A 17-year-old Caucasian female presented to local hospital with a history of acute alcohol intoxication and ingestion of large quantities of acetaminophen. The patient complained of nausea for 2 days and subjective increase in thirst and urine output for a day. She did not notice any dysuria, abdominal pain or change in the colour of her urine. She did however report a remarkable increase in lower back pain that she had attributed to strenuous exercise.

Two days prior to her presentation to the emergency department, the young lady had attended a party where she consumed over 300 mL of vodka and ingested twenty 500 mg tablets of acetaminophen later that night.

Past medical history revealed that she had experienced an episode of rash after having taken liquid acetaminophen as a child. She had not taken acetaminophen since infancy and also denied regular use of any other analgesic. Family history was non-contributory for relevant physiological and psychological illness. The patient reported feeling stressed due to a recent slip in school grades, however denied any suicidal ideation.

Physical examination in the emergency department revealed blood pressure of 140/92 mmHg, pulse 80 bpm, temperature 36.9°C, respiratory rate of 16 breaths per minute and oxygen saturation of 100% at room air. Height and weight were recorded at 170 cm and 63 kg. Physical examination was otherwise unremarkable. No rash, joint swelling or sign of dehydration was detected.

Initial renal function tests at the local hospital revealed serum creatinine and BUN levels of 1.6 mg/dL and 19.3 mg/dL, respectively. Other laboratory results included: glucose 112 mg/dL, sodium 141 mEq/L, potassium 4.3 mEq/L, chloride 104 mEq/L, bicarbonate 29 mEq/L, albumin 43 g/L, aspartate transaminase 20 IU/L, alkaline phosphate 66 IU/L and total bilirubin 1.11 mg/dL. Her INR was reported to be normal at 0.9. Toxicity screens on admission showed acetaminophen level at 9.06 μg/mL. Urine dipstick showed trace amount of blood and 2 plus protein. Microscopy revealed 25 red blood cells per high power field and was otherwise non-contributory. Abdominal ultrasound revealed increased hepatic echogenicity and the kidneys were normal in appearance.

At the outside hospital, she was given morphine, ondansetron and lansoprazole for her back pain and gastrointestinal symptoms. Acetylcysteine was not administered as the patient presented more than 24 hours after the ingestion of acetaminophen.

Renal function tests were repeated later that evening and monitored frequently thereafter. Over the course of 36 hours, the patient's serum creatinine and BUN levels elevated from 1.6 mg/dL and 19.3 mg/dL to 5.7 mg/dL and 49.6 mg/dL, respectively. Urine output was reported to be 2.7 mL/kg/hour. At this time, the patient was transferred to our Children's Hospital for rapidly progressive renal insufficiency.

Physical examination upon admission showed normal vital signs: blood pressure 115/74 mmHg, pulse 60 bpm, temperature 36.6°C, respiratory rate 16 breaths per minute and 100% oxygen saturation at room air. Serum creatinine and BUN levels were measured at 6.3 mg/dL and 52.4 mg/dL, respectively. Furthermore, her electrolytes were recorded as follows: random glucose 86 mg/dL, sodium 134 mEq/L, potassium 5.7 mEq/L, chloride 104 mEq/L and bicarbonate 22 mEq/L. Coagulation study revealed an INR of 1.0 and an APTT of 28 seconds. Other investigations including blood counts and liver functions tests were within normal range. The patient's daily urine output was 3.3 mL/kg/hour at this time. Urinalysis revealed small amounts of protein and blood in her urine. Staining for eosinophils on the random urine sample was positive.

**Figure 1:**
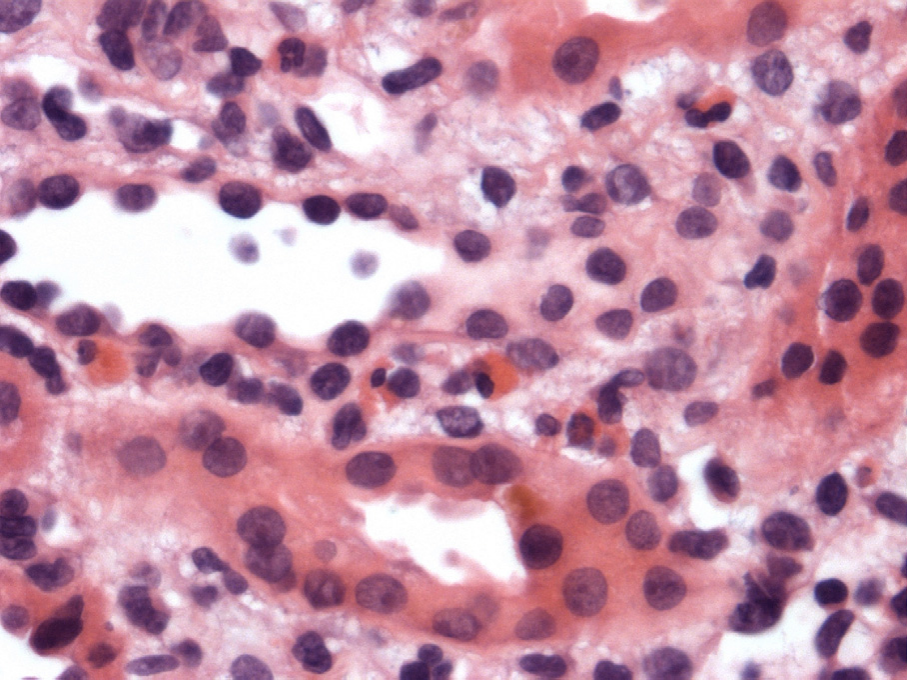
**Renal biopsy of the patient that shows eosinophils infiltration in the interstitium**.

Renal biopsy was performed to further delineate the underlying cause of rapidly progressive renal insufficiency. Figure 1 depicts the histological findings. The biopsy showed no evidence of glomerulonephritis. The interstitium was mildly edematous with small aggregates of inflammatory cells, mainly lymphocytes and eosinophils. Immunofluorescence and electron microscopy were both negative for immune deposits. The histological findings were compatible with acute interstitial nephritis.

The patient was managed supportively by careful monitoring of fluid intake and output. Corticosteroids therapy was considered if no improvement of her renal function was observed. However, her serum creatinine and BUN levels peaked at 7.0 mg/dL and 60 mg/dL two days after admission then began to improve without further medical intervention. Her serum creatinine and BUN levels upon discharge were 4.7 mg/dL and 48 mg/dL respectively. Outpatient follow-up at two weeks after discharge showed that she had completely recovered; her creatinine was 1 mg/dL and BUN was 18 mg/dL.

## Discussion

Drug-induced AIN represents a growing cause of acute renal failure in today's medical practice [1]. This particular report illustrates a case of AIN induced by a widely consumed analgesic that otherwise has little documented correlation with disease pathogenesis. At therapeutic dosages, little evidence suggests that acetaminophen may cause significant harm to liver or kidney. However it is well established that in large quantities, the drug may induce renal and hepatic toxicity leading to tissue necrosis and organ failure [4]. Although this disease presentation differs from what is described in our case (interstitial nephritis), the pathogenesis of this organ failure is important to consider.

It is generally thought that a portion of acetaminophen metabolism occurs via CYP 450 enzymes in both the liver and the kidneys. The resultant reactive metabolites are then reduced by glutathione (GSH) and are excreted as benign compounds in the urine. At higher doses however, more acetaminophen is shunted through these pathways leading to the increased production of reactive metabolites and gradual GSH depletion. The reactive species which are left unbound when GSH is fully depleted may then form adducts with cellular proteins leading to cell death and organ failure. In renal tissues specifically, prostaglandin synthetase and N-deacetylase enzymes are also thought to play a role in reactive metabolite formation and overall disease outcome [6].

In our particular case however, renal biopsy did not reveal tissue necrosis. Instead, the presence of cellular aggregates, namely lymphocytes and eosinophils, as well as peripheral hypersensitivity symptoms (i.e. eosinophiluria) was suggestive of AIN with an allergic etiology. Furthermore, the lack of immunoglobulins and immune complexes indicated a cell-mediated mechanism. With this in mind, it is interesting to note that an established model of drug-induced AIN is a delayed hypersensitivity reaction involving small reactive compounds known as haptens (or pro-haptens) [1]. Essentially, this model suggests that the drug or one of its reactive metabolites may act as a hapten and bind to and modify the immunogenicity of native renal proteins. Consequently, cell-mediated responses targeted to the foreign protein-hapten complexes are initiated [1,7]. This pro-hapten mechanism is what we propose in the reviewed case; where high doses of acetaminophen may have lead to increased reactive metabolite formation and subsequent modification of renal peptides. While this proposed mechanism of acetaminophen-induced AIN is plausible, further research is of course necessary to establish an exact model of pathogenesis. Additional research should also focus on adverse renal outcomes following concurrent acetaminophen and alcohol ingestion.

Our patient received one dose of lansoprazole when she presented to the local emergency room and lansoprazole has also been linked to AIN. In a recent systematic review on proton pump inhibitor (though not specific for lansoprazole) as the possible cause of AIN, among the sixty-four reported cases in literature, the mean duration of treatment before the onset of nephritis was 13 weeks (range: 2 to 52 weeks) [8]. As the patient has already been symptomatic with elevated serum creatinine of 1.6 mg/dL (normal: less than 1.0 mg/dL) before she received her dose of lansoprazole, and only 1 dose was administered, the authors do not think her present illness was related to this medication.

In terms of AIN management, identification and removal of the drug responsible for the pathology is the single most important principle [1,9]. Supportive therapies that include close monitoring of intravascular volume and maintenance of electrolyte balance are also essential. On the other hand, the therapeutic roles of corticosteroids remain controversial. Few large retrospective or prospective controlled studies have been conducted with inconsistent conclusions. While some studies report a more rapid and complete recovery of renal function with steroid administration, others have failed to confirm these results [1,9-11]. In this particular case of acetaminophen overdose however, corticosteroids were not needed, and baseline renal functions were reestablished within 2 weeks time.

As stated, few reported cases of acetaminophen associated AIN exist to date. Those that are reported most often involve patients who suffer from chronic alcoholism [5]. This makes our report an important illustration of acetaminophen-induced AIN with concurrent acute rather than chronic alcohol ingestion.

## Conclusion

Given our findings, we believe that healthcare professionals should be aware of the less recognized adverse affects of acetaminophen overdose. They should also consider AIN in their differential diagnosis when presented with patients suffering from acute renal injury associated with acetaminophen overdose; especially in conjunction with acute or chronic alcoholism.

## Abbreviations

AIN: acute interstitial nephritis; NSAIDs: Non steroidal anti-inflammatory drugs; GSH: glutathione.

## Authors' contributions

The first draft of the paper was written by LF and KL together. IA was responsible for the pathology section. KL was involved in extensive review and revision of the manuscript. The authors have read and approve the final version of this manuscript.

## Competing interests

The authors declare that they have no competing interests.
